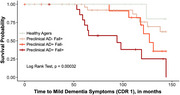# Falls, amyloid positivity, and dementia symptom onset

**DOI:** 10.1002/alz70857_107475

**Published:** 2025-12-26

**Authors:** Audrey A. Keleman, Melody Li, Julie K. Wisch, Rebecca M. Bollinger, Melissa J. Krauss, Elizabeth A Grant, Tammie L.S. Benzinger, Beau Ances, John C. Morris, Susan L. Stark

**Affiliations:** ^1^ Department of Veterans Affairs, Aurora, CO, USA; ^2^ Washington University in St. Louis School of Medicine, St. Louis, MO, USA; ^3^ University of Colorado Denver, Aurora, CO, USA; ^4^ Washington University School of Medicine in St. Louis, St. Louis, MO, USA; ^5^ Washington University School of Medicine, St. Louis, MO, USA; ^6^ Washington University in St. Louis, School of Medicine, St. Louis, MO, USA

## Abstract

**Background:**

In preclinical Alzheimer disease (AD), amyloid accumulates in the brain while individuals remain cognitively unimpaired (Clinical Dementia Rating®[CDR®]=0). Differentiating trajectories of healthy aging and preclinical AD is challenging as both are associated with age‐related impairments (e.g., cognition, motor function, falls).

**Method:**

This longitudinal cohort study investigated the impact of falls and preclinical AD on dementia symptom onset. We prospectively monitored falls for 1 year among 125 CDR=0 older adults and assessed preclinical AD status using amyloid positron emission tomography (PET). We continued to evaluate CDR annually (median 10 years). The cohort was grouped by preclinical AD status (amyloid PET positivity) and fall status (1 or more over the year): Preclinical AD‐Fall‐ (“Healthy Agers”; *n* = 35), Preclinical AD‐Fall+ (*n* = 53), Preclinical AD+Fall‐ (*n* = 16), and Preclinical AD+Fall+ (*n* = 21). Survival analysis examined time to progression to CDR 1 (mild dementia) by group.

**Result:**

Participants were 74 years (mean) at baseline, 62% female, 96% White. Seventy‐four participants (59%) experienced a fall in the year of fall monitoring. Of those who fell, 86% of Preclinical AD+ participants had severe falls (more than one fall or a fall resulting in injury), compared to 53% in Preclinical AD‐ (*p* = 0.01). Preclinical AD+Fall+ progressed to CDR 1 most rapidly. Healthy Agers progressed least quickly. Preclinical AD+Fall‐ and Preclinical AD‐Fall+ had similar progression rates (see Figure). The Cox proportional hazards models revealed that relative to Healthy Agers, participants in the Preclinical AD+Fall+ group had the highest hazard ratio (HR) for progression to CDR 1 (HR = 21.3, *p* < 0.001). Preclinical AD+Fall‐ participants also had a significantly higher risk of progression (HR = 15.1, *p* = 0.01). Falls alone, without preclinical AD pathology, were associated with a moderate, though not statistically significant, increase in risk (HR = 5.6, *p* = 0.08).

**Conclusion:**

Falls may associate with faster progression of AD dementia, potentially reflecting underlying motor and gait dysfunction intrinsic to disease progression. Future research should examine differences in motor function and falls in healthy agers and those with preclinical AD.